# Supplementary material for: Identification of photoperiod-induced specific miRNAs in the adrenal glands of Sunite sheep (Ovis aries)
Source: Front Vet Sci. 2022 Jul 22;9:888207. doi: 10.3389/fvets.2022.888207 (PMC9354845; doi:10.3389/fvets.2022.888207)
Supplement: Supplementary file 1 [file Data_Sheet_1.ZIP › Supplementary file/miRDeep2 file.zip!/miRDeep2 file/Novel_Structure/SPLP42A1/Novel_320.pdf]

Provisional ID	:	NC_019467.2_15323
Score total	:	1.6
Score for star read(s)	:	-1.3
Score for read counts	:	0
Score for mfe	:	1.3
Score for randfold	:	1.6
Score for cons. seed	:	
Total read count	:	31
Mature read count	:	31
Loop read count	:	0
Star read count	:	0

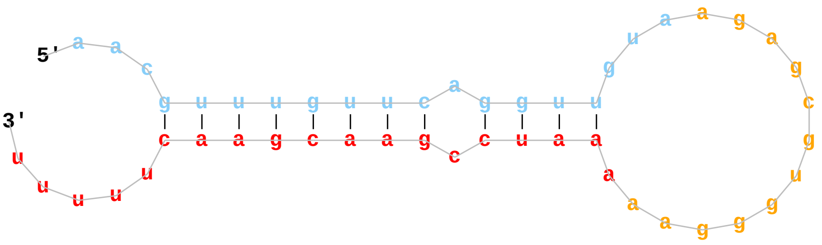

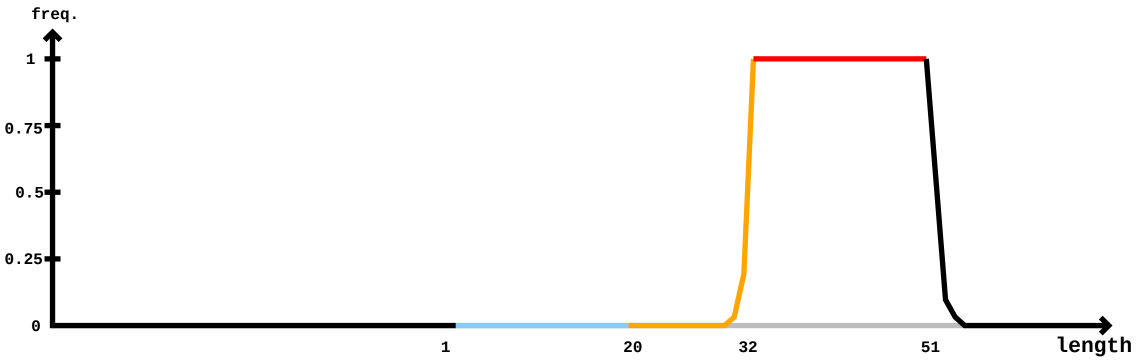


```
                                         Star                 Mature
5'- uggguucuguccuagguaaugguaguacuggguuggccaaaacguuuguucagguuguaagagcgugggaaaaauccgaacgaacuuuuuggccaagucaauaauuug   -3'    exp
    ..(((.(((.((........))))).)))((.((((((((((.((((((((.((((................)))).)))))))).)))))))))).)).........    reads     mm       sample
    .....................................................................aaaaauccgaaUgaacuuuuu..................    1         1        seq
    ......................................................................aaaauccAaacgaacuuuuu..................    3         1        seq
    ......................................................................aaaauccAaacgaacuuuuugg................    2         1        seq
    .......................................................................aaauccgaacgaacuuuuu..................    24        0        seq
    .......................................................................aaauccgaacAaacuuuuuggc...............    1         1        seq
```
